# Miniaturized 3D Depth Sensing-Based Smartphone Light Field Camera

**DOI:** 10.3390/s20072129

**Published:** 2020-04-09

**Authors:** Hyun Myung Kim, Min Seok Kim, Gil Ju Lee, Hyuk Jae Jang, Young Min Song

**Affiliations:** School of Electrical Engineering and Computer Science, Gwangju Institute of Science and Technology, 123 Cheomdangwagi-ro, Buk-gu, Gwangju 61005, Korea; gusaud31@gist.ac.kr (H.M.K.); seok9643@gist.ac.kr (M.S.K.); gjlee0414@gist.ac.kr (G.J.L.); hjjang3472@gist.ac.kr (H.J.J.)

**Keywords:** optical MEMS, miniaturization, light field, 3D depth sensing

## Abstract

The miniaturization of 3D depth camera systems to reduce cost and power consumption is essential for their application in electrical devices that are trending toward smaller sizes (such as smartphones and unmanned aerial systems) and in other applications that cannot be realized via conventional approaches. Currently, equipment exists for a wide range of depth-sensing devices, including stereo vision, structured light, and time-of-flight. This paper reports on a miniaturized 3D depth camera based on a light field camera (LFC) configured with a single aperture and a micro-lens array (MLA). The single aperture and each micro-lens of the MLA serve as multi-camera systems for 3D surface imaging. To overcome the optical alignment challenge in the miniaturized LFC system, the MLA was designed to focus by attaching it to an image sensor. Theoretical analysis of the optical parameters was performed using optical simulation based on Monte Carlo ray tracing to find the valid optical parameters for miniaturized 3D camera systems. Moreover, we demonstrated multi-viewpoint image acquisition via a miniaturized 3D camera module integrated into a smartphone.

## 1. Introduction

Cameras have become indispensable devices for recording human history over the last couple of centuries. Furthermore, research on 3D cameras has been conducted actively over recent years in accordance with the increasing demand for information measurement in the real world beyond simply capturing 2D images. The recent trend of minimizing the size and cost of 3D cameras in many applications, such as smartphones, entertainment, remote sensing for facial recognition, motion detectors, and 3D surface imaging, has motivated research into the miniaturization of 3D cameras [1,2,3,4,5,6]. Moreover, small unmanned aerial systems (sUAS) that include 3D cameras have the potential to offer an anti-collision function and environmental remote sensing; for example, civil and military sUAS with 3D cameras are used in unexpected scenarios during emergencies [7,8,9,10]. However, sUAS pose challenges resulting from their small payload weight and battery capacity, which significantly affects their flight time [9]. These challenges could be overcome by using miniaturized 3D cameras without an additional light source and multi-camera systems. Many 3D camera techniques have been developed, such as stereoscopic vision [11,12,13], structured light [14,15,16], and time-of-flight (TOF) [17,18,19]. Stereoscopic vision systems acquire depth information from two or multiple cameras that capture the same image from different viewpoint angles. Structured light camera systems contain a camera and projector that generate certain geometric light patterns, such as dot arrays, arbitrary fringes, and stripes to perform the 3D reconstruction. TOF cameras are depth-sensing devices that measure the round-trip time of an infrared light signal. However, the aforementioned 3D camera techniques require more than two cameras or external light sources, meaning that they are not suitable for miniaturization. Recent studies on 3D imaging systems have focused on using a single image sensor rather than two identical cameras to reduce the overall size, cost, weight, and battery size of the optical system [20]. As an alternative to the above-mentioned 3D cameras, light field camera (LFC) technology provides suitable conditions and a high potential for miniaturization. They are passive 3D cameras composed of a single image sensor without a light-emitting device [13]. Typically, there are two drawbacks for miniaturizing LFCs: (1) the camera systems are usually large because of the size of the main lens; (2) there are challenges for the focus alignment of the micro-lens array (MLA) in the integration of image-sensor stages.

Here, we report on a novel compact 3D camera based on an LFC consisting of an image sensor, an MLA, and a single aperture to capture different viewpoint-angle images. The size of the LFC optical system was significantly reduced by using only one aperture without the main lens. The aperture and each micro-lens of the MLA act as a camera at different positions, capturing a multi-viewpoint image using a process similar to that in a focused plenoptic camera, which is one of the conventional realizations of the LFC. Optical simulation based on Monte Carlo ray tracing was performed to determine the valid aperture size and distance from the MLA. Furthermore, we propose a simplified integration method to solve the complex optical alignment issue, thus achieving targeted miniaturization. The LFC image-sensor stage was constructed by placing it above the engineered MLA, thereby precluding difficulties in the focus alignment of the micro-unit. Therefore, the proposed compact LFC system addresses the miniaturization issue with both multi-camera and active 3D camera systems.

## 2. Results

**Figure 1:**
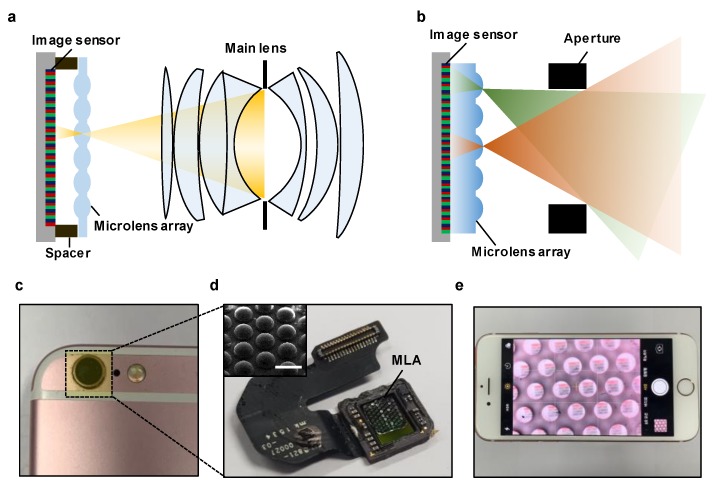
(**a**) Schematic of a conventional light field camera (LFC). (**b**) Schematic of a minimized LFC structure with only one aperture. (**c**) Photograph of a minimized LFC integrated into a smartphone. (**d**) Magnified photograph of a micro-lens array (MLA) stacked on an image sensor. The inset displays a scanning electron microscope (SEM) image of the fabricated MLA. The scale bar is 500 µm. (**e**) Demonstration of the multi-viewpoint image acquisition of the proposed module.

The structure of a conventional LFC comprises the main lens, a micro-lens array, and a single image sensor, as shown in Figure 1a. Generally, LFCs employ a micro-lens array to capture information regarding the intensity and direction of all the light rays from a scene through the main lens [21,22,23,24,25]. Moreover, an alignment structure is used for optical focus alignment at the image-sensor stage to achieve the focal length of the MLA. Such a structure is an obstacle to the miniaturization of LFC systems and to the combination of image sensors with an MLA. We propose a significantly simplified optical system, as shown in Figure 1b, to solve these impediments to the miniaturization of 3D cameras. The scene captured by the single aperture is formed in the image sensor, similar to the multiple camera images captured at various angles through each micro-lens in the micro-lens array. Furthermore, we processed the micro-lens into the form of a plano-convex lens, and the image-sensor stage was configured without an alignment structure to adjust the additional optical focal length through the MLA designed via ray-tracing-based optical simulation. Figure 1c shows a miniaturized LFC system implemented in a smartphone camera using a simple assembly. Note that the focus alignment was achieved by placing the engineered MLA on the image sensor of the smartphone, as shown in Figure 1d. Figure 1e shows a demonstration of image acquisition through a miniaturized LFC configured in the smartphone camera.

**Figure 2:**
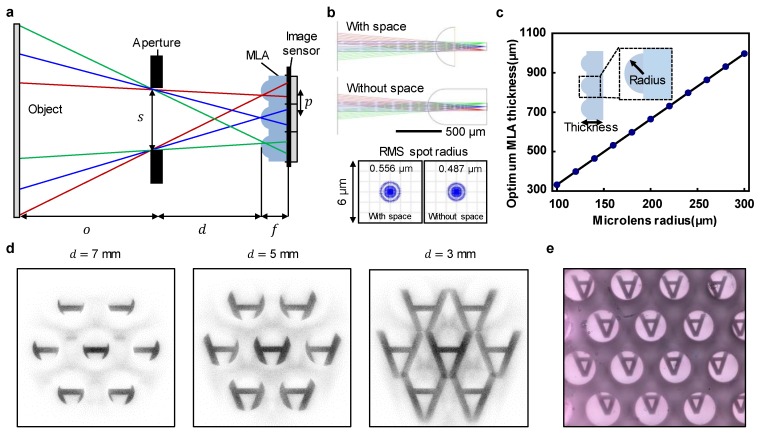
(**a**) Schematic of the analysis parameters for the miniaturized LFC system. (**b**) Schematic of the ray-tracing simulation for a micro-lens with space and without space with the same radius, and comparison of root-mean-square (RMS) spot radii. (**c**) Optimum MLA thickness according to the micro-lens radius. (**d**) Image acquisition simulation results according to the aperture distance from the MLA. (**e**) Multi-viewpoint image acquired using the minimized LFC smartphone camera.

Figure 2a shows a schematic of the relevant parameters in miniaturized LFC systems. The angle formed between the aperture and the micro-lens array produces an image that is taken from various angles of a scene. The angle difference between the captured images is related to the position of the micro-lens and the aperture distance from MLA, denoted by d . The shorter the distance d or the larger the pitch p of the micro-lens, the greater the angle difference between the images. Note that there is an overlap between micro-images. Therefore, it is necessary to determine appropriate values of d and p. We designed a practical micro-lens array pitch p, aperture size s, and distance from the MLA d to achieve this multi-viewpoint image formation. Figure 2b shows the difference in focal lengths with and without space when the micro-lens is composed of poly-dimethylsiloxane (PDMS) with a radius of 200 µm; this value was found from ray-tracing-based simulations. By adjusting the PDMS thickness for the case of a focal length without space, the focal length was relatively increased, thereby reducing the difficulty in controlling the thickness in the manufacture of the MLA [26,27]. Furthermore, by optimizing the thickness of the PDMS, the increased focal length in the absence of space achieved better performance in terms of the root-mean-square (RMS) spot radius. Figure 2c represents simulation data values for an optimal MLA thickness that does not require a space alignment structure according to the radii of the micro-lens. The optimum thickness tends to increase proportionally to the radius. Note that the optimum thickness without the alignment structure can be determined according to the target radius. Our target radius was 200 µm; hence, 0.1 million pixels comprised one micro-image. Moreover, the optimal thickness of the MLA was 665 µm. The areas to consider were the entrance pupil and d. The design value for the entrance pupil of the aperture was 4 mm, which is slightly smaller than the entrance size of the cover glass of the smartphone to allow integration of the LFC system. Figure 2d shows the results of ray-tracing-based image simulation according to the aperture distance from the MLA. When d was set to 7 mm, the field of view of the image coming in through the aperture was small, and the image fill factor tended to decrease significantly for the designed MLA. However, when d was set to approximately 3 mm, the viewing angle became large, and there was overlapping between images. Therefore, we used 5 mm as a valid value for d. As depicted in Figure 2e, the demonstration shows that the captured multi-viewpoint images were well-matched and presented the same tendency as in Figure 2d.

**Figure 3:**
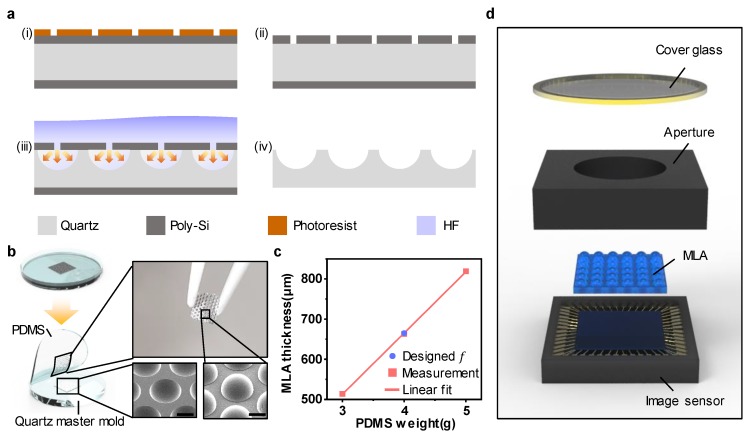
(**a**) Procedure schemes for quartz MLA mold fabrication. (**b**) Schematic of the replica molding process of the poly-dimethylsiloxane (PDMS) MLA. The inset shows an SEM image of the fabricated quartz master mold (left) and replicated PDMS MLA (right). The scale bar is 200 µm. (**c**) The graph shows that the MLA thickness is linearly proportional to the poured PDMS weight in a Petri dish with a diameter of 100 mm. (**d**) A magnified schematic of the configuration of the minimized LFC system.

Figure 3a shows the overall fabrication procedure of the quartz MLA master mold. The fabrication steps were as follows:

(i) For the hard mask of hydrofluoric acid (HF) wet etching, poly-Si was deposited on both sides of a quartz substrate. The thickness of the deposited poly-Si was set to 700 nm to prevent the penetration of HF. Photoresist (PR) hole patterning was performed on one side of the poly-Si using photolithography.

(ii) The patterned sample was dry-etched by using SF_6_ gas through an inductively coupled plasma reactive ion etch (ICP-RIE) to transfer the hole pattern to the poly-Si. The ICP-RIE etching recipe of poly-Si was as follows: SF_6_ flow/working pressure/RF power/ICP power/etching time = 50 sccm/4 mTorr/50 W/100 W/2 min.

(iii) The patterned sample was immersed in an HF bath for 400 min, and the HF solution isotropically etched the quartz through the hole pattern of the poly-Si. The etch rate of the quartz exhibited hemispherical isotropy at the center of each via hole because the via holes were small(diameter: ~ 2 μm). Therefore, micro-lenses were created on the quartz substrate with the same radius and sag height. 

(iv) Subsequently, the poly-Si was removed using potassium hydroxide (KOH) at a temperature of 150 °C for 30 min.

Figure 3b displays the PDMS replica molding process conducted using the fabricated quartz master mold with a concave micro-lens array. An anti-adhesive was sprayed before proceeding with PDMS replica molding. A fluorocarbon mold release agent was used as the anti-adhesive spray (DAIFREE GA-7550, DAIKIN, Japan). The spraying distance from the mold was 30 cm, and the spraying time was 4 s. A PDMS with a density of 0.97 g/cm^3^ was poured into a Petri dish [28]. The optimized MLA thickness was produced by controlling the weight of PDMS using a container and a precision balance [29]. Figure 3c shows the correlation between MLA thickness and PDMS weight (3–5 g); the correlation is almost linear when pouring PDMS in a flat Petri dish with a diameter of 100 mm. The estimated MLA thickness fabricated using PDMS weighing 4g is 663 µm. The designed MLA thickness was achieved with a negligible error of ~ 0.3% compared to the designed focal length of 665 µm. PDMS curing was performed for 6 h at 70 °C in a convection oven. Furthermore, the unleveled top surface of the PDMS MLA prevented proper image formation as a result of focal length misalignment. Thus, the curing of the PDMS MLA must be performed on a leveled optical stage. After the complete curing of the PDMS, the MLA was carefully detached from the quartz master mold. Figure 3d shows an exploded view of the simple configuration of miniaturized LFC systems. The engineered MLA with optimum thickness was placed on the sensor in the sensor module manufacturing stage. Moreover, an aperture of the entrance pupil of 4 mm was fabricated using a 3D printer (Ultimaker, Netherlands, Ultimaker3) to implement the small-factor-form LFC system using a smartphone camera with a simple assembly. Through this manufacturing method, it was possible to achieve miniaturization of the camera in a simple way without making and aligning an elaborate spacer, which is one of the most difficult processes in conventional compact cameras [30,31].

## 3. Discussion

**Figure 4:**
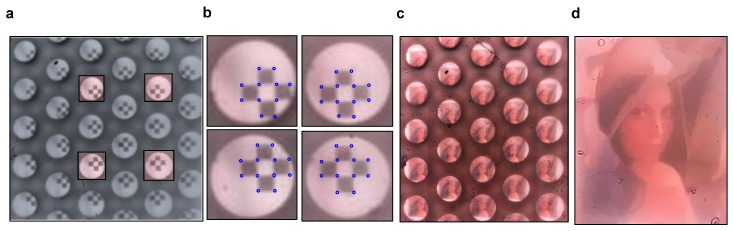
(**a**) Photograph of the checkboard image for calibration with the miniaturized LFC system. (**b**) Reference point image on the checkboard to perform camera calibration. (**c**) Photograph of the Lena picture captured by the miniaturized LFC. (**d**) Images of wider view angles obtained using image stitching techniques from the captured Lena image.

Figure 4 shows how to implement the integration of a stitching image using a multi-viewpoint image, which is one of the essential features of the developed LFC. Figure 4a shows a checkboard image captured with a smartphone light field camera. The captured images show that each micro-image has a distinctly different viewing angle. Typically, camera calibration is performed using checkboard pattern images. To perform the calibration of the smartphone LFC, the reference point was marked on a checkerboard, as shown in Figure 4b. In particular, the reference points were marked on the vertices of each black square in the image. With camera calibration performed in this way, the captured Lena image shown in Figure 4c was processed to the image shown in Figure 4d through the stitching algorithm in MATLAB (Mathworks, USA). Moreover, images with different view directions can be acquired, resulting in wider view angles than the image angles obtained with the center micro-lens. As shown in Table 1, individual micro-lenses had a view angle of approximately 25°, but a stitching image process can be used to obtain images with a view angle of 52°, which is more than twice as wide. By contrast, the main lens of the existing LFC was replaced with a single aperture, and the image was only acquired through a micro-lens, not through a relay optical device. Consequently, there is blur in the images shown in Figure 4. However, the optimization of the micro-lens and the image quality can be improved by minimizing the spherical aberration through an MLA with low sag height [32]. Moreover, many studies have been conducted on thin cameras using MLAs with low effective resolution. Image post-processing methods, such as super-resolution processing, have achieved a level similar to that achieved with the main lens [29,30].

**Figure 5:**
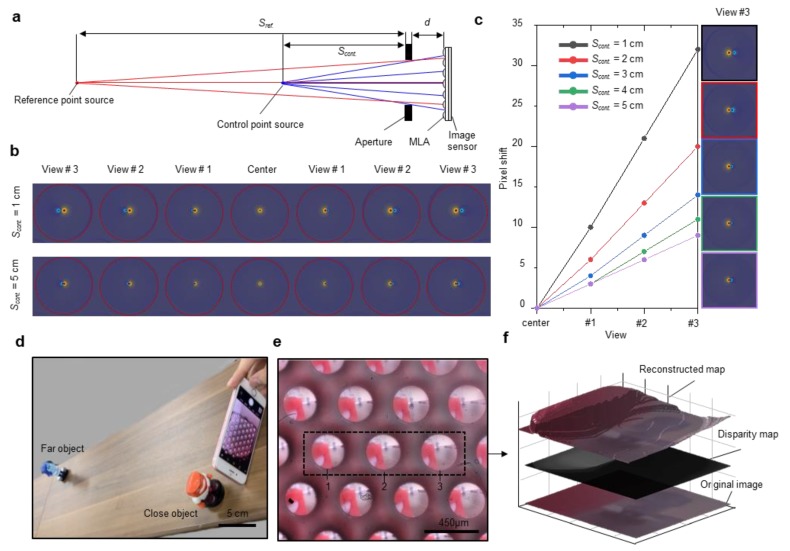
(**a**) Schematic of ray tracing for the calculation of the pixel shift. Parameters *S_ref._, S_cont._*, and *d* represent the reference point source, control point source, and distance between the MLA and aperture, respectively. (**b**) Contour image of the simulation results according to the distance of the point source. Each image shows 7 view-point differences. The red spot depicts the reference point source, whereas the blue spot points out the control point source. In the case of a close distance point source, *S_cont._* becomes 1 cm, and the pixel shift increases (upper case). Otherwise, *S_cont_*_._ is 5 cm, and pixel disparity decreases (bottom part). (**c**) Graph of the pixel shift according to the point source location. (**d**) Photograph of measurement condition. (**e**) The image captured with the mobile light field camera. (**f**) Post-processed image with the obtained light field image. Each layer represents the original image (bottom layer), disparity map (middle layer), and reconstructed map (top layer).

Figure 5 shows a comparison of simulation and measurement results for the quantitative analysis of pixel disparity according to the change in distance due to different view angles in the image. A schematic diagram for performing a ray-tracing-based simulation of a designed optical system that can measure point sources located on the same optical axis is shown in Figure 5a. Such sources cannot be measured with a conventional camera for a pixel shift against distance changes. The distance *S_ref._* of the reference point source was set to 1 m, and the simulation result was obtained by moving the distance *S_cont._* of the control point source from 1 to 5 cm. Figure 5b shows that the pixel shift for the distance change tends to increase as the object gets closer to the aperture than the reference point and as the micro-image gets farther from the center micro-lens. Figure 5c shows a quantitative pixel shift according to the control source point distance change for the image obtained from the center to view # 3 for each micro-lens position. The pixel shift exhibits a linear change because the change in the viewing angle changes linearly with the position of the micro-lens.

To carry out multi-viewpoint image acquisition, raw data were obtained by placing a near object (3 cm) and a distant object, as shown in Figure 4d. The raw data were captured, as shown in Figure 4e, and the centers of the images in the black dotted boxed area were set as the reference line to analyze the difference in the view direction between the micro-image arranged on the left and on the right. The angles of the viewpoint vary depending on the images formed by the respective micro-lens. The lateral pixel position difference between the distant and near object image pixels was compared to analyze the disparity of these images. Table 2 shows the pixel point position for each image based on the remote reference line. The data in parentheses represent the shift in the lateral pixel position between adjacent viewpoints. In adjacent views, the lateral shifts of the red object are 6 pixels. The pixel change of the red object was the same as that in simulations in which *S_cont._* was located at 3 cm. In contrast, the pixel shift was zero because the blue object was located on the remote reference line. Figure 5f represents the post-processed image with the obtained multi-viewpoint image. To demonstrate 3D imaging, we extracted the disparity map by using a local stereo matching method [33]. Each viewpoint image in the obtained raw data was cropped and aligned with a distant blue object, which serves as the reference point source in Figure 5a. Then, disparity maps were extracted using aligned images with the same size through local stereo matching (Figure 5f, middle layer). In addition, reconstructed images were extracted using disparity and cropped images (Figure 5f, top layer). Consequently, we successfully demonstrated multi-viewpoint image acquisition by a single aperture, which is the most important feature for the validation of the 3D depth-sensing function of our miniaturized LFC system.

## 4. Conclusions

In summary, the results reported in this paper demonstrate that miniaturized 3D camera systems in smartphones based on light field cameras offer several attractive features, including compact configuration. These features can be implemented in conventional smartphones, passive depth-sensing systems that do not require significant energy consumption or a large volume of active lighting, resilient technologies consisting of one image sensor without camera synchronization, and compensation of single-sensor deviations. The miniaturized 3D camera design is implemented using different viewing directions. It comprises a single aperture and a micro-lens array. This compact system design was theoretically validated using ray-tracing-based simulations. Moreover, a sequential fabrication process (i.e., photolithography, isotropic wet etching, polymer replica molding, and 3D printing processes) was used to implement the engineered micro-lens arrays and apertures. Multi-view image acquisition and 3D depth map extraction, which are key elements of light field cameras, were also achieved with captured images using a compact 3D camera system integrated into a smartphone. The results indicate that miniaturization of the proposed 3D camera system, along with its simplified optical configuration, lighter weight, and lower power consumption, is a promising path toward advanced versions of compact depth-sensing systems for electronic applications that are being downsized.

## Figures and Tables

**Table 1:** Specifications of the smartphone light field camera.

Pixels of each sub-image	260 × 260
Radius of the micro-lens	200 µm
Diameter of each micro-lens	400 µm
Focal length of each micro-lens	665 µm
Acceptance angle of each micro-lens	25°
The total field of view	52°

**Table 2:** Pixel location of the red and blue objects from the reference point in Figure 5e.

Object	View-1	View-2	View-3
Red	48	42 (6)	36 (6)
Blue	15	15	15
